# Supplementary material for: Neutrophil-generated HOCl leads to non-specific thiol oxidation in phagocytized bacteria
Source: eLife. 2018 Mar 6;7:e32288. doi: 10.7554/eLife.32288 (PMC5839695; doi:10.7554/eLife.32288)
Supplement: Transparent reporting form [file elife-32288-transrepform.docx]

***eLife’s* transparent reporting form**

We encourage authors to provide detailed information *within their submission* to facilitate the interpretation and replication of experiments. Authors can upload supporting documentation to indicate the use of appropriate reporting guidelines for health-related research (see [EQUATOR Network](http://www.equator-network.org/%20)), life science research (see the [BioSharing Information Resource](https://biosharing.org/)), or the [ARRIVE guidelines](http://www.plosbiology.org/article/info:doi/10.1371/journal.pbio.1000412) for reporting work involving animal research. Where applicable, authors should refer to any relevant reporting standards documents in this form.

If you have any questions, please consult our Journal Policies and/or contact us: [editorial@elifesciences.org](mailto:editorial@elifesciences.org).

**Sample-size estimation**

- You should state whether an appropriate sample size was computed when the study was being designed
- You should state the statistical method of sample size computation and any required assumptions
- If no explicit power analysis was used, you should describe how you decided what sample (replicate) size (number) to use

Please outline where this information can be found within the submission (e.g., sections or figure legends), or explain why this information doesn’t apply to your submission:

This information does not apply to this submission:

- There was no *a priori* power analysis performed, since it is typically not necessary for the type of experiments we conducted.

- N/A

- We based the number of repetitions, typically triplicates of biologically independent samples (for definitions see below section “Replicates”), on our, and others, experience with bacterial and mammalian cell culture. This is work under highly controlled and replicable conditions, very different from animal- or patient-based studies. Information on this can be found in the “data analysis” section of the Materials and Methods section.

**Replicates**

- You should report how often each experiment was performed
- You should include a definition of biological versus technical replication
- The data obtained should be provided and sufficient information should be provided to indicate the number of independent biological and/or technical replicates
- If you encountered any outliers, you should describe how these were handled
- Criteria for exclusion/inclusion of data should be clearly stated
- High-throughput sequence data should be uploaded before submission, with a private link for reviewers provided (these are available from both GEO and ArrayExpress)

Please outline where this information can be found within the submission (e.g., sections or figure legends), or explain why this information doesn’t apply to your submission:

This information can be found in the “Data analysis” part of the materials and methods section.

- 96-well co-incubation assays were repeated at least three times with biologically independent replicates.

- Biologically independent replicate: PLB-985 cells, as well as bacteria were grown and differentiated independently, often on different days. Technical replicate: The same bacteria population was incubated with the same PLB-985 cell population, however in separate wells in one or more 96-well plates.

- We provide data as outlined in section “source data”

- After establishment of our final experimental conditions, no outliers were detected.

- N/A

- N/A

**Statistical reporting**

- Statistical analysis methods should be described and justified
- Raw data should be presented in figures whenever informative to do so (typically when N per group is less than 10)
- For each experiment, you should identify the statistical tests used, exact values of N, definitions of center, methods of multiple test correction, and dispersion and precision measures (e.g., mean, median, SD, SEM, confidence intervals; and, for the major substantive results, a measure of effect size (e.g., Pearson's r, Cohen's d)
- Report exact p-values wherever possible alongside the summary statistics and 95% confidence intervals. These should be reported for all key questions and not only when the p-value is less than 0.05.

Please outline where this information can be found within the submission (e.g., sections or figure legends), or explain why this information doesn’t apply to your submission:

This information is found in the data analysis section of the materials and methods section.

- Statistical methods were generally confined to calculation of the mean and standard deviation of multiple biological and technical replicates, as described in the section “replicates”. Since the key findings are typically differences in oxidation kinetics of the probes used, a Student’s T-test or other statistical measurement of end-point data is not applicable.

- Although N < 1, presentation of raw data directly in the figure would not be informative. We provide the raw data separately as outlined in “source data”.

- Values of N for each individual experiment are found in the “source data”.

- N/A

(For large datasets, or papers with a very large number of statistical tests, you may upload a single table file with tests, Ns, etc., with reference to sections in the manuscript.)

**Group allocation**

- Indicate how samples were allocated into experimental groups (in the case of clinical studies, please specify allocation to treatment method); if randomization was used, please also state if restricted randomization was applied
- Indicate if masking was used during group allocation, data collection and/or data analysis

Please outline where this information can be found within the submission (e.g., sections or figure legends), or explain why this information doesn’t apply to your submission:

These procedures are described in the materials and methods section, however most of this information does not apply to this submission.

- Typically bacterial cultures were split and the very same cultures then treated with external stressors (such as H2O2) or differentiated, non-differentiated or treated PLB-985 cells. Similarly, for our experiments with inhibitors or stimulants, PLB-985 cell cultures were split and the same cultures were then treated with inhibitors, stimulants or were differentiated.

- No masking was used.

**Additional data files (“source data”)**

- We encourage you to upload relevant additional data files, such as numerical data that are represented as a graph in a figure, or as a summary table
- Where provided, these should be in the most useful format, and they can be uploaded as “Source data” files linked to a main figure or table
- Include model definition files including the full list of parameters used
- Include code used for data analysis (e.g., R, MatLab)
- Avoid stating that data files are “available upon request”

Please indicate the figures or tables for which source data files have been provided:

Figure 1 D, E, F, G, H, I, J

Figure 2 A, B, C, D

Figure 3 G, H, I

Figure 4 A, B, C

Figure 5 A, B, C, D, E

Figure 7 A, B

Figure 8

Figure 9 A, B, C

Figure 10 A, B, C, D, E
